# Oral health goes to school

**DOI:** 10.1080/07853890.2021.1897366

**Published:** 2021-09-28

**Authors:** Rozan Cecilia, J. Carmo, A. Peixoto, A. G. Manso

**Affiliations:** a Centro de Investigação Interdisciplinar Egas Moniz (CiiEM), Egas Moniz Cooperativa de Ensino Superior, Caparica, Portugal; b Instituto Universitário Egas Moniz (IUEM), Egas Moniz Cooperativa de Ensino Superior, Caparica, Portugal

## Abstract

**Introduction:**

Preventive dental care is an essential component of comprehensive health care and early epidemiological diagnosis plays a crucial role in the secondary prevention of oral disease [1,2]. The aim is to characterise oral hygiene habits, to measure dental plaque and gingivitis.

**Materials and methods:**

On behalf the "VI edition of the health fair of Alhos Vedros", promoted by UCSP (Personalize Self-Care Unit) of Alhos Vedros – Portugal in 2018, with the theme "Health goes to school". Resorted to basic and disposable observation material, we observed a population composed with children, adults and elderly people and registered the presence of visible plaque and gingivitis (Figures 1–3). Subjects were answered two questions about oral hygiene habits. The sample was obtained by convenience and treated descriptively by the prevalence of cases.

**Results:**

Twenty-nine people were observed, aged 5–77 years. 79% of individuals brushed their teeth twice or more times daily, with a higher brushing frequency among the younger population. 65.5% of the individuals brushed their teeth with toothpaste, and 34.5% used toothpaste associated with a mouthwash (without plaque developer). 76% of the population observed had visible plaque, and more than half were children aged between 5–14 years. 48.3% of the population had gingivitis, most frequently on the adult population (68.4%), compared to 31.6% in the population aged 5–14 years (Figures 1–3).

**Discussion and conclusions:**

The youngest population has higher prevalence values for brushing and bacterial plaque. The prevalence of gingivitis was higher in the adult population. The use of bacterial plaque developer may benefit the oral hygiene of this population.

**Figure 1.**
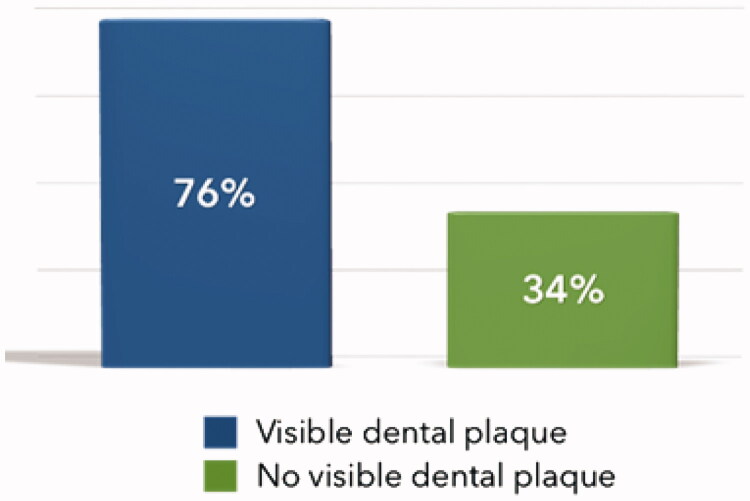
Prevalence of visible dental plaque.

**Figure 2.**
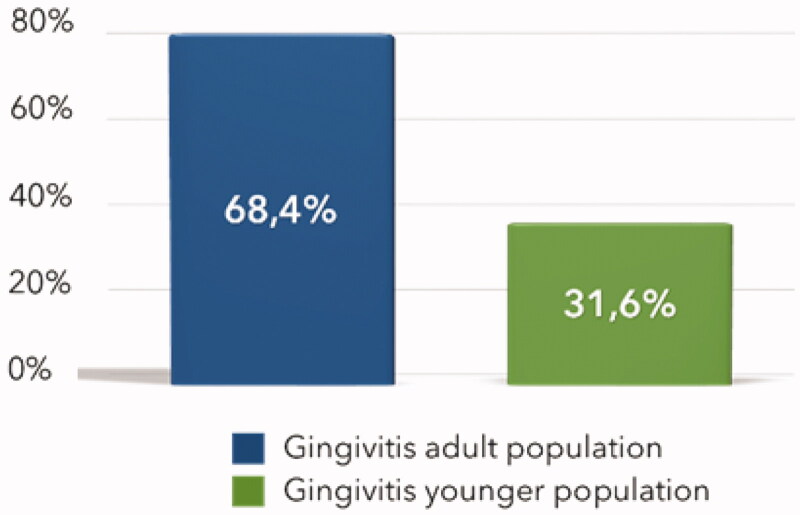
Prevalence of gingivitis among the population.

**Figure 3.**
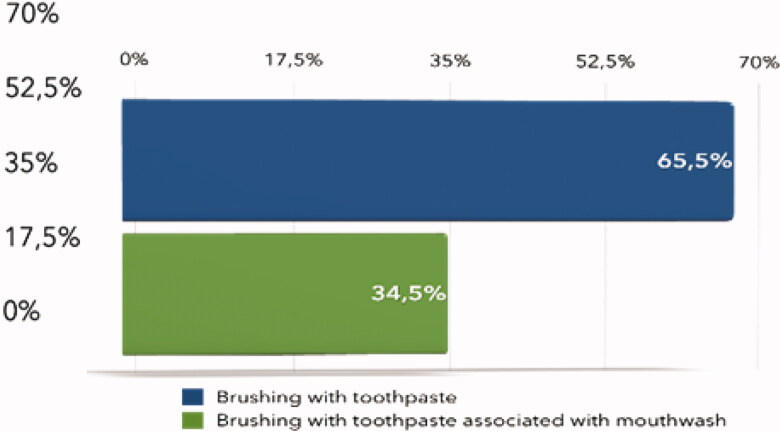
Prevalence of oral hygiene habits among the population.
